# Aerosols generated by high-speed handpiece and ultrasonic unit during endodontic coronal access alluding to the COVID-19 pandemic

**DOI:** 10.1038/s41598-022-08739-3

**Published:** 2022-03-21

**Authors:** Mirela Cesar Barros, Victor Feliz Pedrinha, Evelyn Giuliana Velásquez-Espedilla, Maricel Rosario Cardenas Cuellar, Flaviana Bombarda de Andrade

**Affiliations:** Department of Operative Dentistry, Endodontics and Dental Materials, Bauru School of Dentistry, University of São Paulo, Alameda Dr. Octávio Pinheiro Brisola, 9-75, Bauru, São Paulo 17012-901 Brazil

**Keywords:** Microbiology, Dental diseases, Infectious diseases, Oral diseases

## Abstract

To investigate the dispersion and contamination of aerosols generated during coronal access performed by high-speed handpiece and ultrasonic device. To measure the aerosol dispersion, a red dye or an *Enterococcus faecalis* culture broth inside the bottle of the water system of the dental and ultrasonic unit were used. Bovine extracted teeth were allocated in six groups according to the coronal access: G1: diamond bur in high-speed handpiece (HS) with aspiration (A); G2: ultrasonic (US) inserts with aspiration; G3: combined coronal access with HS and US with aspiration; and G4, G5, and G6 were performed without aspiration (WA). The distance reached by the aerosol with the dye was measured in centimeters, and for environment contamination, agar-plates were arranged at standardized distances for counting colony-forming units (CFU/mL). The ANOVA followed by the Tukey tests were applied (*α* = 0.05). The coronal access with HS generated higher aerosol dispersion and contamination, even with simultaneous A (*P* < 0.05), while US generated less aerosol even WA (*P* < 0.05). The aspiration did not reduce the aerosol statistically. HS is a great source of aerosols in dental clinic during the coronal access and the use of US device should be encouraged.

## Introduction

Aerosols generated during dental procedures have become a target of concern and discussion^1,2^, since are vectors of infective agents including bacteria, yeasts, filamentous fungi, and viruses^3–7^. So, aerosol particles show high potential for contamination that not only reach the dental care professional and patients, but also all exposed surfaces of the dental unit and the operatory environment^6,7^. Not different, there is a risk of transmission of acute respiratory syndrome coronavirus 2 (SARS-CoV-2), which results in the COVID-19 disease, in dental practice carried by aerosol particles^2,3^ from blood, saliva, and other body fluids exposure of symptomatic and asymptomatic patients, combined with instruments that generate aerosols^8^.

Routine procedures such as cavity preparations, ultrasonic devices and especially the use of air/water jets being the main responsible for the dispersion of aerosol particles^9–12^. Regarding the SARS-CoV-2, is estimated that aerosolized particles remain in environment for 3–16 h^9,13^. Thus, a high contaminated dental clinic environmental can be considered a cause of infection^1–3^. At the beginning of the pandemic, recommendations were adopted to avoid procedures that generate aerosols^9^. With the increase in the number of positive cases for COVID-19, asepsis measures in dental offices have become more stringent than in the past^14,15^. However, there is a lack of knowledge about disinfection protocols and how to perform them, in addition to the behavior of the SARS-CoV-2 virus on surfaces and substrates by the dental practitioners^16^. It is necessary to pay attention and make professionals aware of the need to adopt disinfection measures in the office environment and the use of personal protective equipment.

Studies in the medical field suggest the use of devices that reduce aerosol dispersion. However, they do not consider the needs of dental practice^17,18^. Therefore, there is a need for studies that evaluate, especially in the pandemic context, the amount of aerosol produced and its dispersion during a coronal access performed in endodontic treatment^1^. Technological advances in the dentistry field have allowed devices such as ultrasonic inserts to be routinely used in various endodontic procedures, including coronal access^19^. The E6D and E7D (Helse Ultrasonic, Santa Rosa de Viterbo, SP, Brazil) ultrasonic inserts were recently introduced in endodontics aiming to perform the coronal access and pulp chamber refinement. To the best of our knowledge, no studies have investigated the aerosol dispersion and dental clinic environment contamination generated by ultrasonic inserts during the coronal access. Some studies were limited to restorative and periodontal procedures^1,2^. To date, the high-speed system is the most used device for coronal access, and it produces a considerable amount of aerosol^20^.

Alternative strategies aiming to reduce the aerosol dispersion as well as the reduction of dental clinic environment contamination need to be investigated, also considering the possibility of reducing the chances of the SARS-CoV-2 dissemination and also others pathogenic microorganisms. With this background, this in vitro study aimed to compare the aerosol dispersion and the dental clinic environment contamination produced by the high-speed system and the E6D and E7D ultrasonic inserts, during coronal access, with or without simultaneous high aspiration suction. The null hypothesis was that coronal access performed with the different strategies promotes aerosol dispersion and dental clinic environment contamination in a similar way.

## Materials and methods

All methods were carried out in accordance with relevant guidelines and regulations. The sample size was calculated using the G*Power v 3.1 software for Mac (Heinrich Heine, University of Düsseldorf, Germany), selecting the test comparing more than 2 means for independent groups (ANOVA). Alpha type error of 0.05, beta power of 0.85 and N2/N1 ratio of 1 were also stipulated. The test showed a total of 20 samples for each group as an ideal size to observe significant differences.

A single operator with expertise in the endodontic field performed the coronal opening procedures to ensure that the bur and/or ultrasonic insert contact with the tooth was continuous. To standardize coronal opening cavities, 120 bovine maxillary incisors were opened from the central area of palatal/lingual side to start the surgical access. Beyond the esthetic purpose, it also represents the shortest path to the pulp chamber. The election point to access for each tooth was created using a fine-tip black marker. The access was extended from the cingulum to within 2 mm of the incisal edge to expose the entire pulp chamber cervico-incisally and mesio-distally, according to recommended literature^21^. The teeth were positioned inside a dental model, on a dental chair, in a reclined position, simulating a clinical treatment situation. The operator worked at the 11 o'clock position using personal protective equipment, including face shield, following the recommendations for dental care during the pandemic period^9^.

### Experiment 1: Dispersion (cm) of the produced aerosol

**Figure 1:**
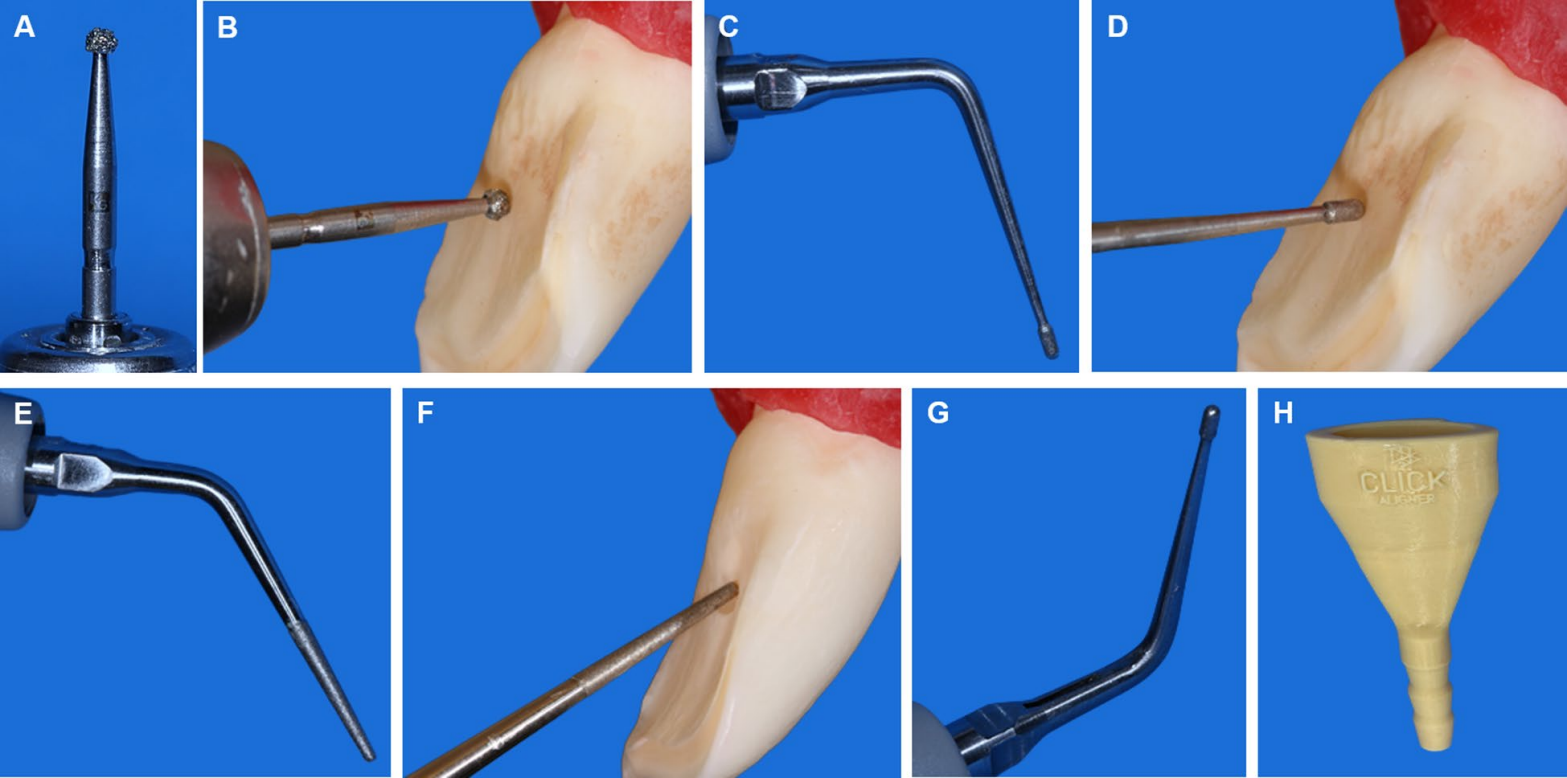
(**A**) The 1014 diamond bur connected to a high speed handpiece to access incisors; (**B**) a representative image of direction of access surgery performed with diamond bur; (**C**) the E6D ultrasonic insert; (**D**) a representative image of direction of access surgery performed with E6D ultrasonic insert; (**E**) the E7D ultrasonic insert; (**F**) a representative image of access cavity refining moment; (**G**) the ultrasonic insert water system arrangement; (**H**) the aspiration sucker device employed in the experiments.

To measure the distance reached by the aerosol, the dental chair was covered with white TNT and a red food coloring dye (Arcolor, São Paulo, SP, Brazil) was added to the water outlet of both the ultrasonic device and the dental unit water line. Then, the specimens were inserted into a dental model and allocated according to the coronal opening strategy: use of the 1014 HL spherical diamond bur (KG Sorensen, Cotia, SP, Brazil) coupled in a high-speed (KaVo do Brasil Ind.Com.Ltda., Joinville, SC, Brazil), and the E6D and E7D diamond inserts (Helse, Santa Rosa do Viterbo, SP, Brazil) coupled in an ultrasound (Acteon, Mount Laurel, NJ, USA) with a potency of 8% (Fig. 1). During pilot studies, a single operator performed the same procedures of coronal opening at several times without using the red food coloring dye to standardize the time for coronal access using a chronometer. It was found that the average time for each coronal access was approximately 3 min, considering bovine teeth that are much larger than human teeth. Both systems were used with maximum opening of the water outlet to standardize the water volume and to enable the greatest possible amount of aerosol produced by devices evaluated. After coronal opening, the distance reached by the dye was measured with a metal measuring-tape.

### Experiment 2: Evaluation of the contamination produced by the systems

In a similar way to experiment 1, coronal openings were made, however, an inoculum of *Enterococcus faecalis* (ATCC 29212) was placed in the water bottle of the devices to supply contamination. The strain was reactivated in BHI broth (Difco, Kansas City, MO, USA) and kept at 37 °C for 24 h. Then, the bacterial culture was transferred to another flask and incubated for another 24 h in order to reach its exponential growth. Culture purity was confirmed by colonial morphology and Gram stain (Oxoid, Basingstone, UK) in an optical microscope at 1000× magnification (Olympus Europe CoGmbH, Hamburg, Germany) throughout the experiment. A 12-h growth was standardized for medium contamination.

**Figure 2:**
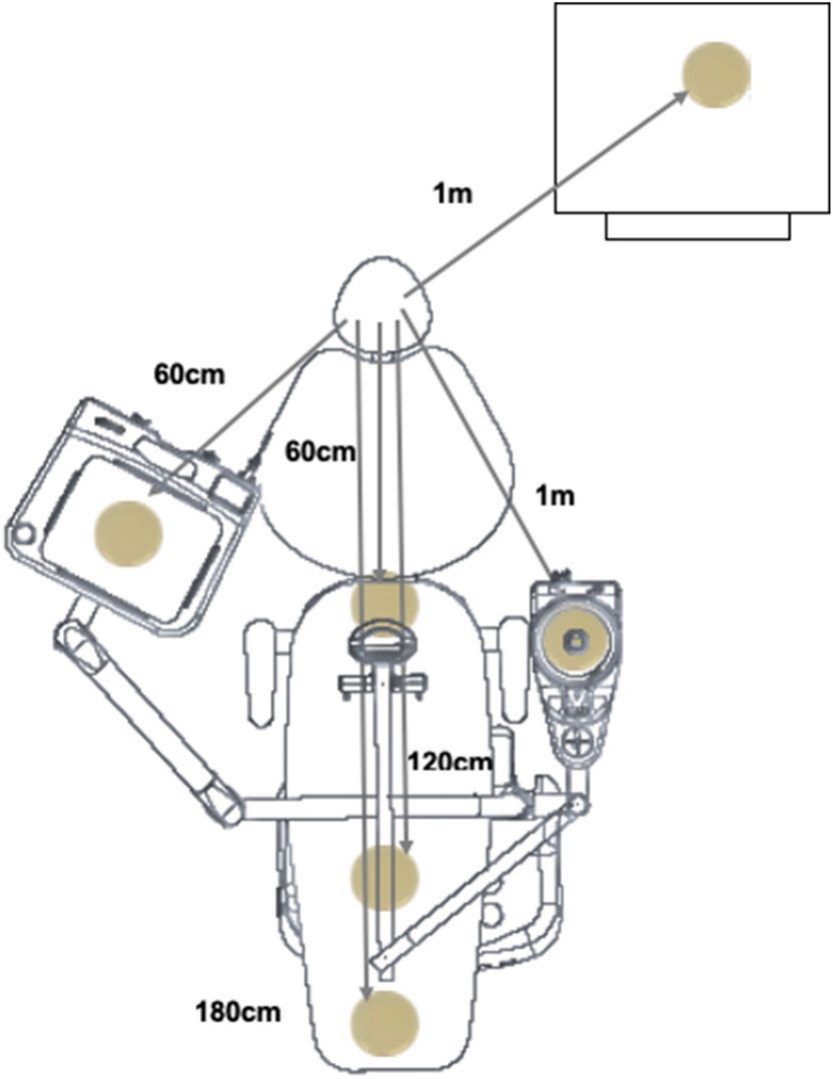
Positioning the BHI agar plates at distances of 60, 120, and 180 cm from the coronal access point and three additional plates, one on the right side at 60 cm, one on the left side at 1 m and other behind the operator at a distance of 1 m.

For each specimen, six BHI agar plates without their lids were placed on the dental chair, three linearly at distances of 60, 120 and 180 cm from the access point and three additional plates, one on the right side at 60 cm, one on the left side at 1 m and other behind the operator at a distance of 1 m, placed on standardized supports (Fig. 2). After the coronal openings, the plates were closed and incubated in a bacteriological incubator for 48 h for a subsequent counting of colony forming units (CFU/mL). In order to confirm the absence of contamination in the environment, serving as a negative control, plates containing BHI agar were placed in the same positions 24 h before the beginning of the experiments.

### Experimental groups

The specimens (teeth) were allocated into six experimental groups (N = 20). Ten teeth of each group were opened with the dye and the other 10 were opened with the *E. faecalis* culture in the water bottle of the devices. The groups allocated according to the device for coronal access were:

- G1: High speed (HS) without aspiration (WA)
- G2: Ultrasound (US) − WA
- G3: US + HS − WA
- G4: HS with aspiration (A)
- G5: US − A
- G6: HS + US − A

In the groups with simultaneous aspiration, an extra-oral dental aspirator device (3D LAB—Anycubic photon, Betim, MG, Brazil) was used coupled to a portable aspiration pump (Nevoni—5005BRST, Barueri, RS, Brazil), simulating a clinical situation. This aspirator device was developed aiming a higher aerosol aspiration in dental clinic and less dispersion of contaminated droplets. Moreover, it shows a larger suction chamber opening (Fig. 1). A calibrated auxiliary operator positioned the aspirator device immediately in front of coronal opening access point.

### Statistical analysis

Data were expressed as mean and standard deviation. After the Shapiro–Wilk test to verify the normality, data were analyzed by analysis of variance (ANOVA) to assess differences between groups, followed by Tukey's t test for multiple comparisons using a 5% significance level.

## Results

### Aerosol dispersion

In all groups tested, there was aerosol dispersion, from 22.56 to 72.30 cm of distance, on average. The longest point was 87 cm from the source, produced by the HS group, without aspiration. The use of high speed even associated with simultaneous aspiration promoted a greater dispersion of the aerosol generated at a distance greater than or equal to 60 cm (Table 1).

**Table 1:** The mean and standard deviation values of aerosol dispersion measured in centimeters by the tested groups.

Groups	Aerosol dispersion (cm)	
	Mean	Std. deviation
High speed (HS) without aspiration (WA)	72.30^a^	7.98
Ultrasound (US) − WA	29.50^b^	5.96
US + HS − WA	63.44^d^	2.78
HS with aspiration (A)	22.56^a^	7.41
US − A	48.50^c,b^	15.57
HS + US − A	42.40^e,d^	5.77

Different superscript letters indicate a significant difference between groups (P < 0.05).

**Figure 3:**
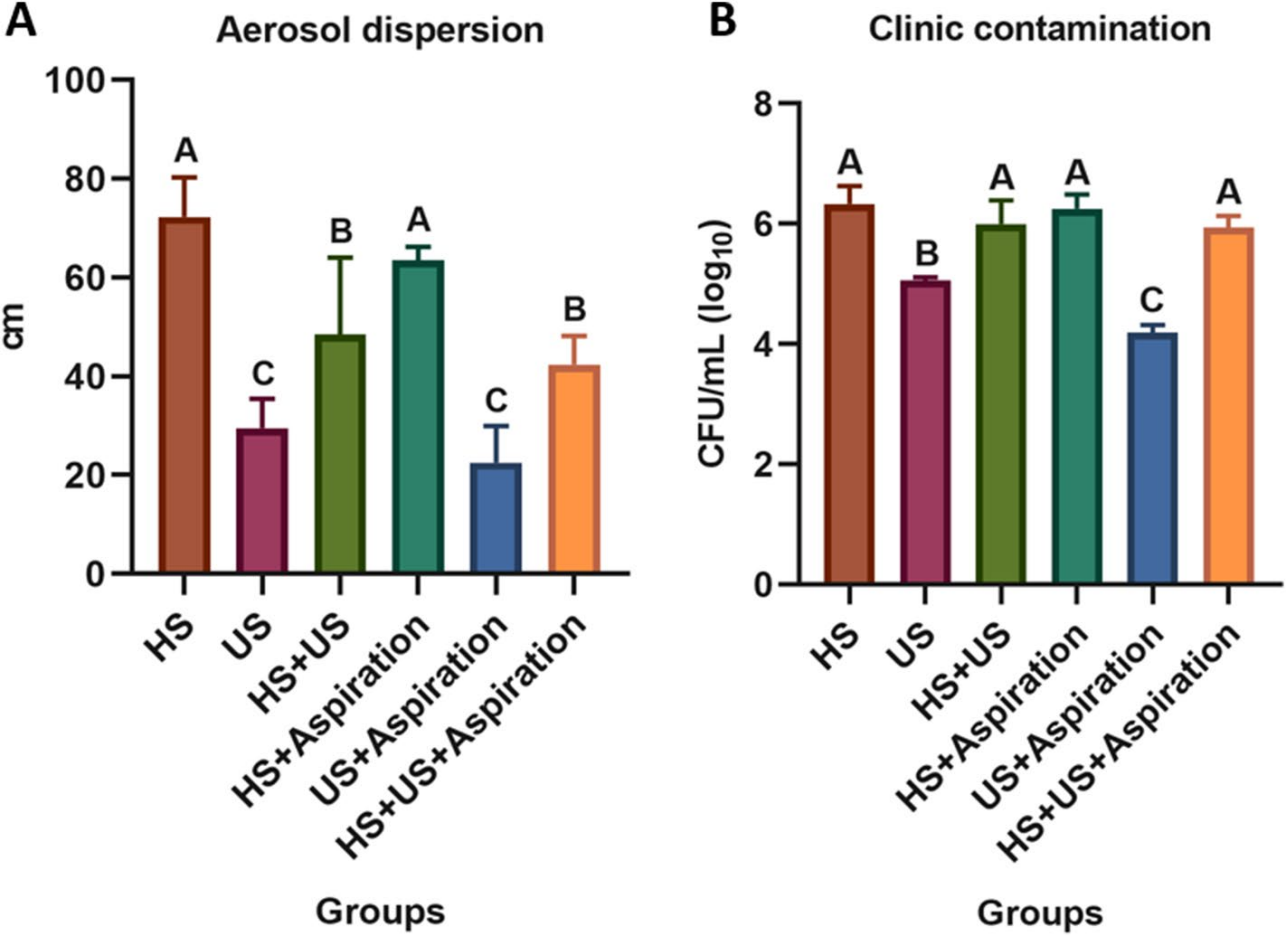
(**A**) Measurement of the distance in centimeters reached by the aerosol produced by introducing red dye to the AR and US water system; (**B**) Bacterial growth (CFU/mL) produced by the aerosol by introducing the inoculum into the AR and US water bottles.

HS, US and HS + US were statistically different among each other (*P* < 0.05). However, no differences were detected when using or not the aspiration (Fig. 3A).

Dye stains were seen on the operator's head, chest, right arm, and face shield. The operator was fully clothed with protective material and was not at risk of being exposed to contamination. Although all systems have produced aerosol, when using ultrasound to perform coronal openings, the aerosol dispersion was smaller even without the use of aspiration.

### Contamination produced by the systems: high speed and ultrasound

There was bacterial growth in all groups, confirming the results observed in experiment 1 (Fig. 3B). Even with simultaneous aspiration, the use of high speed generated a greater amount of aerosol, a fact that can be observed when using it alone or in association with ultrasound. Groups 2 and 5 showed statistical differences (*P* > 0.05) compared to the others, where contamination was considerably lower even without the use of aspiration (Table 2). In this particular situation, the use of aspiration could contribute for a less contamination when using only the ultrasonic device, being statistically different.

**Table 2:** Data of the environmental contamination produced by the systems. Comparison by ANOVA and Tukey’s post hoc tests.

Groups	Log_10_ (CFU/mL)	
	Mean	Std. deviation
High speed (HS) without aspiration (WA)	6.32*	0.28
Ultrasound (US) − WA	5.06	0.04
US + HS − WA	5.99*	0.37
HS with aspiration (A)	6.25*	0.22
US − A	4.18	0.12
HS + US − A	5.93*	0.18

*No statistically significant differences.

## Discussion

In this study, methodologically, the enamel and dentin tissue wear phase preceding the real fall into the pulp chamber were performed with a spherical diamond bur and/or two ultrasonic inserts. In a dental clinic and especially in a pandemic context, the use of rubber dam is mandatory in dental procedures^3^. However, the present work aims to use strategies to maximize aerosol dispersion. In addition, the rubber dam prevents observing the position of the tooth in the simulated dental arch that can lead to the occurrence of perforations during the coronal opening^17,22^. Moreover, bovine incisors present different crown dimensions than human teeth crowns. If rubber dam had been used in this essay, maybe few bacteria would likely have been present, complicating comparison of the two access techniques.

To investigate contamination, the sampling method with "seating plates" was used, which quantifies viable bacteria through plates containing culture medium^8,18,23^. Plates were strategically positioned in places that are part of dentist's field of action during clinic procedures in order to assess the reach and contamination of the aerosol generated from the devices used. Also, plates were positioned on the dental chair to assess the risks offered to the patient. A bacteria inoculum was added to the water bottles of both devices evaluated to mimic the contaminated environment of the oral cavity. *Enterococcus faecalis* was chosen as a biological tracer, a facultative anaerobic bacterium, due to its characteristics of commonly being isolated from persistent endodontic infections^24^. Despite dentists and their team are constantly exposed to the highly contaminated environment of the oral cavity, which presents a varied microbiota^3^, in the present study a single specie was used aiming to perform a true standardization of the infection source.

It is important to evaluate a bacterium contamination and dispersion to attempt to the danger of cross infection through aerosols. In fact, even before the SARS-CoV-2 pandemic, the potential airborne spreading of life-threatening infections was well recognized^18^. A classic example is the study of Miller et al. (1971), however, they did not use a tracer microorganism like in the present study^5^. In a previous study, *Streptococcus mutans* was used as a biological tracer infused into the mouth of a manikin to simulate the diffusion of any infective agent by aerosol^7^. This previous investigation attempted to minimize or avoid the use of rotary and ultrasonic instruments when concerns about airborne spread of pandemic disease agents as SARS-CoV-2 are present^7^. In the present study, two methods were performed for aerosol evaluation: the contamination (CFU/mL) and the mapping of aerosol dispersion area (cm). Since there is a lack of investigations regarding aerosol particles and preventive measures or techniques to reduce aerosolized microorganisms generated during endodontic coronal access, comparisons are required as much as possible to provide consistent scientific evidence.

Contamination and maximum dispersion were observed when using high speed, regardless of simultaneous aspiration, in the region of the operator's arms, chest, and face shield, followed by the dental chair and other places in the office at a distance of up to 87 cm. Previous studies have shown similar results showing that in addition to these regions, areas around the nose and inner corners of the eyes had a significantly higher rate of contamination^8,25^. Regarding the level of contamination, this was significantly higher in the groups in which high speed was used, with a mean count value of 6.0 (± 0.2) Log_10_CFU/mL, in agreement with other studies that evaluated it during dental and endodontic care^3,23^. On the other hand, Harrel and Molinari demonstrated that the ultrasound, even without water system produced a significant aerosol dispersion when liquid was placed at the operation site, simulating blood and saliva^26^. According to our findings, despite the aerosol production, the amount, distance, and contamination were considerably lower for ultrasound device. Inclusively, the use of aspiration could contribute for a less contamination when using only the ultrasonic device (Fig. 3). These differences in the results may be correlated with the lack of standardization in the power of the ultrasonic device and in the design of the water outlets between inserts from different manufacturers.

Further to the use of personal protective equipment a new extra-oral aspirator device developed for the pandemic scenario was used in groups with aspiration. In a clinic condition, regardless of the care adopted, dentists are at greater risk of contamination by the COVID-19 virus and other pathogen transmissions within the office and outside it^3,7^. In this context, strategies that aim to manage waste, minimize exposure, and disinfection of the office environment are extremely important. Studies show that dentists understand the importance of disinfection protocols, but there is lack of knowledge on how to perform them, in addition to the behavior of the SARS-CoV-2 virus on surfaces and substrates. Even so, professionals are receptive to the adoption of these protocols, and there is a need to improve this topic worldwide, through lectures and/or training so that they can proceed with their activities in a safe way for the team and patients^16,17,27–29^.

It is accepted that small particles with a diameter of < 5–10 μm that follow the airflow are potentially capable of short and long-range transmission. Particles < 5 μm easily penetrate the human airways into the alveolar space and particles of < 10 μm below the glottis^11,30,31^. In this context, masks must be used in a well-fitting way, as contaminants can bypass the filtering effect of these, entering through their pores. Studies show that the proper fit and positioning of the mask, the operator's movement, as well as the voice level when speaking have a direct influence on the efficiency of bacterial filtration^32^. It has been shown that the severe acute respiratory syndrome (SARS) virus is easily spread through the aerosol due to its particle size (5–10 μm), and the same is true for the SARS-CoV-2 virus due to their structural similarity^33^. *Enterococcus faecalis,* once dispersed in the air, manages to remain viable, contaminating the environment. In order to preserve a maximum bacteria viability, the plates were immediately closed by the assistant after coronal openings and taken to an oven at 37 °C by 48 h until the CFU/mL counts. This method performed in the present study is commonly used and allows for the cultivation and counting of viable bacteria, which therefore have the potential for contamination^4,5,7^.

The literature points out that the aerosol remains for at least 3 h after the procedure, spreading and contaminating surfaces^13,34,35^. Therefore, it is recommended that the operator, as well as the assistant, do not remove the protective barriers immediately after the procedure, reducing the risk of contact with airborne contaminants. Environmental disinfection methods using lamps that emit 250–265 nm ultraviolet radiation are effective, however, they are costly and not accessible to all professionals^36^. In this context, more economical and also effective techniques, such as simultaneous vacuum aspiration during procedures involving the generation of aerosol, the use of rubber dam isolation to perform the coronal access, and encouragement to the use of tools that guarantee the same results, such as the ultrasound device, should be strongly stimulated^37,38^.

As this is an in vitro study, some limitations include the fact that the air quality assessment could not be demonstrated, as the tests were performed in a simulated model of a dental office. In addition, the coronal access was made only in anterior teeth by high rotation and/or ultrasonic device, so the results should not be generalized as they may be different for posterior teeth. However, the present work provides an alternative for performing the coronal opening and opens the way for the applicability of ultrasonic inserts associated, according to our results, with lower aerosol formation. Added to this, the risks of aerosol formation can be minimized by following simple and inexpensive precautions: use of adequate ventilation in the office environment, disinfection of surfaces between appointments, use of protective glasses, face shield and high-power aspiration devices^7,8,17,36^.

In conclusion, the use of high speed, alone or in conjunction with other systems, is one of the main factors for generating aerosols in the dental office, responsible for the risk of contamination. In endodontics, the step for using high speed, is the coronal opening and due to the lower potential for contamination, the use of ultrasound should be encouraged, especially accompanied by high power aspiration, considering known and emerging pathogens such as SARS-COV-2.

## Data Availability

All data generated or analyzed during this study are included in this published article.
